# High-Fructose Diet–Induced Hyperuricemia Accompanying Metabolic Syndrome–Mechanisms and Dietary Therapy Proposals

**DOI:** 10.3390/ijerph20043596

**Published:** 2023-02-17

**Authors:** Michalina Lubawy, Dorota Formanowicz

**Affiliations:** Department of Medical Chemistry and Laboratory Medicine, Poznan University of Medical Sciences, 60-806 Poznan, Poland

**Keywords:** uric acid, metabolic syndrome, fructose, diet

## Abstract

Fructose is often used as a food ingredient due to its low production costs and sweetening power. In recent years, it has been noticed that people on a Western diet high in fructose have high levels of uric acid in their blood. It was recognized that the specific metabolism of fructose in the body might cause increased production of uric acid, which then may affect the intensification of lipogenesis and the development of metabolic syndrome (MetS), insulin resistance, gout, cardiovascular diseases, leptin resistance, or non-alcoholic fatty liver disease. So far, to treat hyperuricemia, it has been recommended to use a low-purine diet characterized by limiting protein-containing products. However, this recommendation often leads to an increased intake of carbohydrate-rich foods that may contain fructose. Increased fructose consumption may enhance the secretion of uric acid again and, consequently, does not have therapeutic effects. Therefore, instead of a low-purine diet, using healthy diets, such as DASH or the Mediterranean diet, which can benefit metabolic parameters, could be a better proposal. This article provides an overview of this approach, focusing on MetS and hyperuricemia among high-fructose dieters.

## 1. Introduction

Many specialists are aware that patients can harm themselves with an improper diet. They generally recommend following the principles of a healthy lifestyle, such as eating a large amount of fruit and vegetables, limiting the consumption of fats, sugar, and salt, and physical activity, regardless of coexisting diseases [1]. However, not all medical doctors and nutrition specialists follow the latest guidelines for individual disease entities, which may make their health endeavors ineffective. One such example is issuing general recommendations for a low-purine diet for patients with hyperuricemia and MetS features without avoiding high-fructose products. It should be noted that specialists should pay much more attention to the amount of fructose in the diet. The recommended daily fructose intake is not entirely defined; however, 25–50 g a day is often indicated as safe, 50–100 g a day as high, and over 100 g daily as dangerous for the human organism [2,3].

Pure fructose and some products containing it, such as sugar, high-fructose corn syrup, and processed foods, are increasingly appearing on our tables [4,5]. They seem to play a significant role in increasing uric acid (2,6,8-trioxypurine, UA) synthesis in the body, which may be crucial for many pathologies [6]. UA is an organic compound formed in the liver, secreted mainly by the kidneys and intestines. The UA concentration in the blood depends mainly on diet and endogenous synthesis [7,8]. Hyperuricemia is diagnosed when the level of UA in the blood is elevated. For pre-menopausal women, the limit is 6 mg/dL; for men and postmenopausal women, it is 7 mg/dL [7,9].

Accumulated evidence from epidemiological studies suggests that hyperuricemia is directly associated with diseases such as hypertension, diabetes mellitus, morbid obesity, hyperlipidemia, and kidney failure [4,10,11,12] and is linked to MetS.

MetS comprises a cluster of metabolic abnormalities, including visceral obesity, insulin resistance (IR), glucose intolerance, hypertension, and atherogenic dyslipidemia. The presence of visceral obesity has been shown to be a significant trigger for most of the pathways involved in MetS, highlighting the importance of high caloric intake disproportionate to metabolic requirements as a major causative factor. As MetS is quickly becoming a global health problem that increases the risk of developing cardiovascular diseases and type 2 diabetes, the issue is attracting more and more research attention.

In our article, we tried to investigate the following: (1) gathering information on the mechanism of hyperuricemia induced by a high-fructose diet and its relation to the MetS syndrome and (2) determining what should be the diet of patients with hyperuricemia and metabolic syndrome in the light of current knowledge.

This review was intended to summarize our current knowledge of hyperuricemia, and a high-fructose diet, mainly focusing on possible treatment among MetS sufferers. In finding relevant articles, we relied on both our own experience and the PubMed search engine for various combinations of the terms “uric acid”, “hyperuricemia”, “low purine diet”, “and fructose.” The recent 10 years of studies and reviews, as well as a review of references of the included studies, were taken into account in this study. The list of all included studies was manually searched separately by two authors to identify new and significant studies that might merit inclusion.

Based on the selected articles and our experience, we discussed the mechanisms that may interfere with the proper diet therapy of hyperuricemia and gout using a traditional low-purine diet. In particular, we emphasized the effect of a high-fructose diet on UA levels and parameters related to the MetS, which often coexists with hyperuricemia. We also presented possible dietary management in patients with hyperuricemia and MetS based on the Mediterranean diet and the DASH diet (Dietary Approaches to Stop Hypertension), which seem to be a better solution than the traditional low-purine diet. Finally, we showed an example of possible dietary recommendations based on the case reports of two patients.

## 2. Link between Hyperuricemia and Metabolic Syndrome

Criteria of MetS do not mention elevated serum UA concentration, although a growing body of research suggests a close relationship between these two conditions [13,14,15,16,17,18,19,20,21,22]. A cohort study by Cicero et al. [14] proved that hyperuricemia predisposes the development of MetS. In addition, these authors noted that, according to their findings, the UA cut-off values associated with the risk of MetS were far lower than those used for the diagnosis of gout. In turn, a meta-analysis evolving 900,000 patients by Rahman et al. [23] showed a significant relationship between the existence of urolithiasis and the presence of MetS features. Li et al. [16] showed in their studies that higher levels of UA are associated with MetS, both in women and men. They also indicated that pre-menopausal women are at greater risk of developing MetS than post-menopausal women. Nagahama et al. [18] pointed out that the risk of developing MetS increases with increasing UA concentration. In studies examining the inverse relationship, both the studies of Besiroglu et al. [21] and Rams et al. [22] showed that patients with MetS have an increased risk of urolithiasis and, thus, higher levels of UA.

According to previous studies, serum UA levels are significantly associated with a history of lipid disorders. After matching for sex, age, income, and region of residence, gout patients were more likely to have a prior history of dyslipidemia than healthy controls. This study revealed a link between gout and dyslipidemia, and the results confirm the detrimental effect of lipid disorders on gout [24].

In a large prospective population study of African Americans and middle-aged white adults, hypertensive patients were twice as likely to develop gout. The relationship between hypertension and gout was independent of gout risk factors, including renal function [25].

Another cross-sectional study showed that each 1 mg/dL increase in serum UA concentration contributed to a 20% increase in the prevalence of hypertension in the general untreated hyperuricemic hypertensive population [26].

There are a few mechanisms whereby UA can cause hypertension. The crystal-dependent mechanisms include (1) the activation of the intrarenal renin–angiotensin–aldosterone (RAA) system; (2) the deposition of urate crystals in the urinary lumen; and (3) direct endothelial injury and dysfunction. Crystal-independent mechanisms include (1) endothelial dysfunction by reducing endothelial nitric oxide synthase phosphorylation under hypoxic conditions and (2) soluble UA upregulating the expression of aldose reductase in the endothelium and other tissues, resulting in the activation of the polyol pathway, leading to the blockade of nitric oxide production and the production of endogenous fructose. Both of these mechanisms seem to play an important deleterious role in the pathogenesis of high blood pressure in the endothelium as the blockade of aldose reductase or fructokinase, the enzyme involved in the metabolism of fructose, markedly improves endothelial cell function (it has been shown that UA induces mitochondrial dysfunction and superoxide generation through the activation of nicotinamide adenine dinucleotide phosphate (NADPH) oxidases, thus depleting energy (adenosine triphosphate (ATP)) capacity [27].

Piani et al. drew attention to the possibility of gout-associated nephropathy caused by microcrystalline deposits, which may be an unknown cause of chronic kidney disease [28]. Researchers also pay attention to the pro-oxidative mechanisms of UA that link hyperuricemia with cardiovascular disease. Among them, they mention others: inhibition of vasodilation, inhibition of adiponectin synthesis, generation of a chronic inflammatory reaction, or activation of the renin–angiotensin system, vascular smooth muscle cell profiling, and angiotensin production [29].

**Figure 1:**
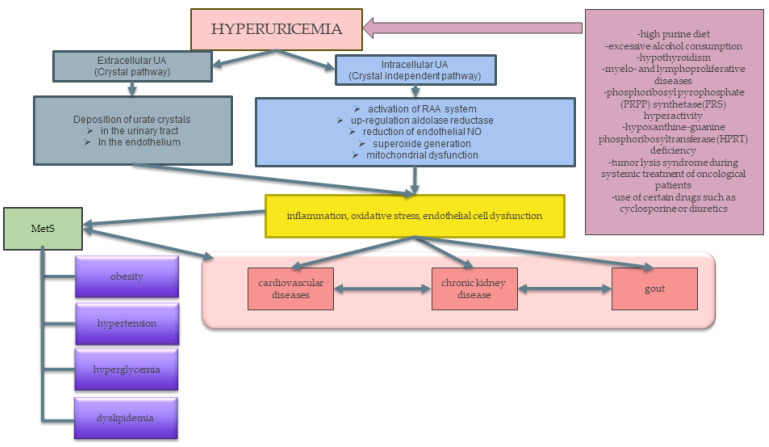
The relationship between hyperuricemia and MetS. Factors such as a high purine diet, high alcohol consumption, certain diseases, consumption of certain drugs, or metabolic disorders lead to an increase in the level of uric acid in the blood, which then causes an increase in inflammation, oxidative stress, and epithelial cell dysfunction. This leads to the development of MetS-related illnesses and diseases, gout, cardiovascular disease, and chronic nephritis. Abbreviations: MetS—metabolic syndrome; RAA—renin–angiotensin–aldosterone; NO—nitric oxide.

The relationship between hyperuricemia and MetS is shown in Figure 1.

Hyperuricemia can be both a cause and a result of other disorders. It can be divided into symptomatic, characterized by clinical signs of gout, and asymptomatic, without clinical signs of gout but with symptoms that may be related to MetS [30]. Therefore, it can be concluded that the relationship between hyperuricemia and MetS is bidirectional. Conditions associated with symptomatic and asymptomatic hyperuricemia are presented in Table 1.

## 3. Fructose

Fructose is a monosaccharide commonly found in food, primarily fruits, vegetables, and honey. It is also a sweetener and ingredient in high fructose corn syrup (HFCS), often used due to its low production costs and high sweetening power. Changes in the agri-food industry in recent years have increased the amount of fructose consumed from 16–24 g to even around 80 g per day in the case of average in the United States. It is estimated that 330–380 kcal consumed daily by Americans comes from fructose, which accounts for 17–20% of daily energy consumption [4,5].

Until recently, the effect of fructose was considered beneficial or neutral [34]. Its glycemic index is 32, much lower than pure glucose or sucrose, even though it has a much higher sweetening power. Fructose also does not produce a significant insulin surge [4].

Studies have shown the adverse effect of fructose on the occurrence of hyperuricemia and gout [35]. It is worth noting that fructose is present in food products in various forms. The literature often raises the issue of whether fruit-derived fructose can contribute to hyperuricemia and gout [11]. An important aspect of these considerations is the fact that fruits, apart from fructose, also contain many other health-promoting compounds, such as vitamins, flavonols, fibers, or trace elements that can modify the adverse effects of fructose [36]. A meta-analysis by Ayoub-Charette et al. [37] showed that there is no statistically significant relationship between fruit consumption and gout. However, an unfavorable relationship between gout and fruit juice consumption has been demonstrated. The literature lacks studies evaluating which fruit juices affect the occurrence of hyperuricemia and gout. A cohort study by Choi et al. [38] showed that fruit juices high in fructose may increase the risk of gout. Other studies have shown that consuming only orange juice was associated with a higher incidence of gout [39]. Research by Kanbay et al. [40] proved that not only the amount of fructose consumed matters, but also the speed of its consumption. They note that fructose consumption from solid foods tends to take longer than that of a beverage and recommend that liquids be consumed in small portions to reduce the damaging effects of fructose. Studies also indicated the negative impact of sugar-sweetened beverages on UA levels [41,42,43]. The researchers also noted high consumption of fructose-containing products such as glucose-fructose syrup and fructose-glucose syrup. Excess fructose consumption may increase the level of UA in the blood due to the characteristic fructose metabolism (stimulation of the catabolism of adenine nucleotides) [4].

## 4. Fructose as a Potential Factor Influencing Hyperuricemia and Metabolic Syndrome

Fructose metabolism is similar to glucose metabolism; however, there are some differences. Two glucose transporters, GLUT 5 and GLUT 2, are mainly involved in fructose absorption [44,45]. Some researchers also mention the SLC2A9 transporter [2]. Fructose is absorbed through the brush border of the small intestine into the enterocyte by GLUT 5 and then transferred into systemic circulation by GLUT 2 [46]. Its absorption mainly takes place in the liver via fructolysis, but also includes the intestinal epithelium, renal proximal tubule, adipocytes, and possibly vascular endothelium [2,46]. In cells, it is mainly metabolized by fructokinase (KHK) to produce fructose 1-phosphate. In contrast to glucose, whose metabolism is involved in phosphorofructokinase, KHK does not have a negative feedback system. As a result, with an oversupply of fructose, ATP can be depleted leading to phosphate depletion, activation of AMP deaminase, and UA production. In addition, fructose metabolism is not hormonally regulated and is independent of insulin [2,40]. In an article by Nakagawa et al. [47], the authors hypothesized that dietary fructose and endogenously synthesized fructose may cause various diseases through its ability to induce the Warburg effect. UA formed during its metabolism is a potential factor causing the process of metabolizing glucose to lactate by cancer cells (Warburg effect) with the help of fructose. The role of UA in this fructose metabolism is that UA can prevent the targeting of fructose metabolites with mitochondrial oxidation. This was discovered using the HepG2 human hepatocellular carcinoma cell line [47]. As a consequence, glucose metabolism is disturbed and leads to cell proliferation, amino acid production, protein synthesis, sufficient energy and, consequently, inflammation. A comparison of glucose and fructose metabolism is shown in Table 2, based on [2,47,48].

When fructose breaks down, glucose, lactate, FFA, UA, methylglyoxal, and triglycerides (TG) are produced. High TG levels are probably due to increasing fatty acyl coenzyme A and diacylglycerols [2]. In research, Johnson et al. [49] have shown that fructose consumption is associated with a decrease in urine pH. This is explained by the increased burden on the kidneys by lactic acid, which is a metabolite of fructose [50].

High levels of fructose metabolites can cause various conditions, especially those belonging to the components of the MetS and hyperuricemia often included in it [46].

**Figure 2:**
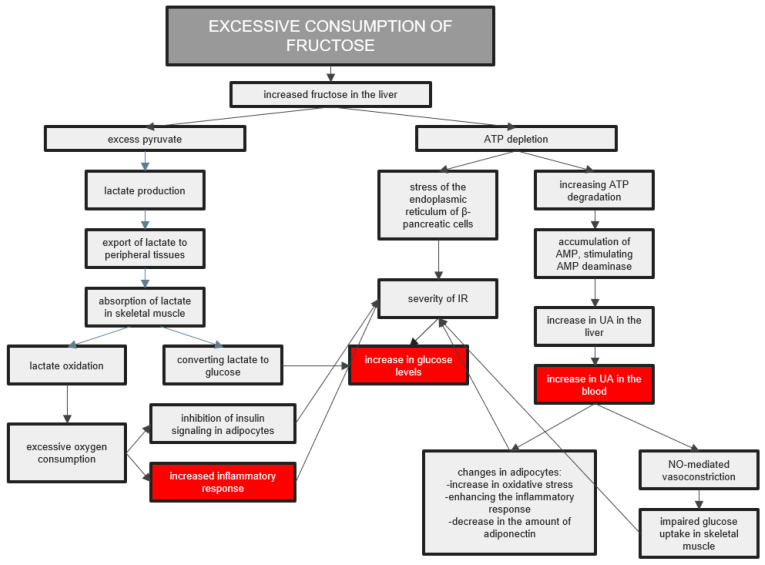
The body’s reactions to excessive fructose consumption. The increased amount of fructose in the liver leads to an inflammatory reaction in the body, increasing glucose and uric acid levels in the blood. Abbreviations: IR—insulin resistance; UA—uric acid.

Fructose is converted to glucose when its consumption is moderate. At the same time, high fructose consumption leads to excessive GLUT 5 induction, increasing the concentration of fructose in the cytosol of the intestinal epithelial cells [45]. In addition, ATP depletion occurs during fructose metabolism, which leads to inflammation and oxidative stress. It causes disruption of the functioning of some tissues and organs and the excessive production of pro-inflammatory cytokines [46]. In addition, UA is produced during ATP depletion, which increases intestinal permeability and exacerbates lipogenic processes in the liver [51]. The body’s reactions to excessive fructose consumption are presented in Figure 2.

Studies by Stanhope et al. [52] proved that, in contrast to glucose, fructose administered in the form of a drink increases TG levels, especially at night. Cohen and Schall [53] compared the effects of glucose, fructose, and sucrose on TG concentration. It turned out that pure glucose did not affect lipid levels, which led to the conclusion that fructose, alone or in the form of sucrose, had such an effect. It follows that fructose can influence the concentration of TG, the level of which is the criterion of MetS.

An excessive supply of fructose causes an increase in the concentration of UA in the blood, which may lead to the development of hyperuricemia. Studies also indicated that fructose may have the ability to shift water intracellularly, causing high serum osmolarity, and consequently lead to low urine output and increase the risk of urate stones [49,54]. Research conducted by Choi et al. [41] on a very large group of over 14,000 people indicated that the consumption of sweetened soft drinks is associated with higher levels of UA. It is also worth noting that such a relationship was not observed in the case of consumption of diet soft drinks that do not have added sweeteners containing fructose.

Additionally, IR caused by consuming excessive amounts of fructose may increase UA acid concentration by reducing its excretion from the body and increasing inflammation, e.g., by secreting tumor necrosis factor α (TNF-α) and interleukin-1β (IL-1β) [11,46]. The increased UA levels caused by excessive fructose consumption can lead to endothelial dysfunction and further to the development of cardiovascular disease. In combination with a high salt intake, fructose causes an increase in blood pressure by impeding the renal reabsorption of sodium [46].

Research by Johnson et al. [2] showed that urinary UA excretion in rats fed with fructose was reduced. As an explanation for this phenomenon, they suggested that it may be due to hyperinsulinemia, lactate production, and hyperuricemia, which led to endothelial dysfunction and renal vasoconstriction.

Leptin is a hormone secreted by adipocytes and is involved in the energy balance of the organism and weight regulation. Its amount is proportional to the amount of adipose tissue and increases with weight gain. In the case of obesity caused by improper diet, high levels of leptin no longer fulfill their role and do not inhibit weight gain. This phenomenon is called leptin resistance [55]. In studies conducted on rats, Shapiro et al. [55] and Chotiwat et al. [56] proved that large amounts of fructose in the diet can induce leptin resistance. One of the reasons for this phenomenon is that the excessive amount of circulating TG inhibits the transport of the hormone through the blood–brain barrier. As mentioned before, it has been proven that the high-fructose diet increases blood triglyceride levels, which suggests its possible influence on the induction of leptin resistance and, thus, overweight and obesity. In addition, it is worth noting that fructose used as a flavoring substance increases the taste of food products, and thus may increase appetite, as well as lead to addictive behavior by stimulating dopaminergic pathways [57].

The study by Béghin et al. examined the effect of consuming fructose from various dietary sources, both natural (e.g., vegetables, fruits, or honey) and industrial (e.g., cakes, confectionery, or sugar-sweetened drinks), on the level of diastolic blood pressure in young girls aged 12.5–17.5 years. It turned out that unlike fructose from natural sources, the one from industrial sources raises the value of blood pressure [58]. Studies in rats fed water enriched with fructose showed that increasing the amount of this sugar in the diet caused hypertension, hyperuricemia, and hypertriglyceridemia in the animals. Glomerular hypertension and renal hypertrophy were also observed [59].

Fructose increases lipogenesis and blocks the oxidation of β-fatty acids, which leads to fat accumulation in the liver, leading to non-alcoholic fatty liver disease (NAFLD) [51]. Studies show that NAFLD patients are often characterized by elevated TG values, low HDL-C, overweight or obesity, excessive blood pressure, and abnormal fasting glucose levels [60]. NAFLD is increasingly being said to belong to MetS, as is hyperuricemia.

The significance of fructose consumption on blood glucose levels is not fully understood, as the studies show many differences depending on others’ age or the administered dosage. Fructose indeed induces changes in the metabolism of substrates in the liver; still, it is not unequivocally confirmed its effect on the overall amount of glucose produced. Fructose does not directly contribute to insulin secretion by the pancreas. Still, its impact on the sweet taste receptors in β cells may cause increased insulin secretion stimulated by glucose, leading to IR [61].

The researchers also pay more and more attention to the influence of high UA concentrations on the development of diabetic nephropathy. The fact that fructose is produced endogenously in diabetes by the polyol pathway leads to the formation of urinary effluvium, which may further damage the interstitial tubules [62].

**Figure 3:**
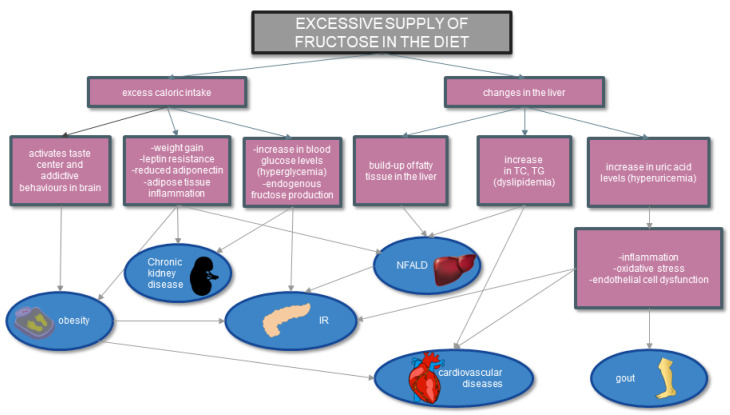
Effect of fructose on metabolic diseases based on [8,45,63]. Excessive consumption of fructose leads to both increased caloric intake and changes in the liver. As a result of such an incorrect diet, there are a number of changes in the body that lead to obesity, IR, NFALD, cardiovascular diseases, or gout. Abbreviations: IR—insulin resistance; TG—triglycerides; TC—total cholesterol; UA—uric acid; NFALD—nonalcoholic fatty liver disease.

The association of fructose consumption with specific diseases and conditions is illustrated in Figure 3.

Moreover, research suggests that a diet rich in fructose also affects the immune system and may disturb the body’s immune homeostasis through, for example, changes in the intestinal bacterial flora and the impact on the permeability of the intestinal barrier. Attention is paid not only to the role of fructose itself, but also to the impact of its metabolites on the immune system [64] Fang et al. [65] drew attention to the effect of fructose on the intestinal microflora and the possibility of fructose-inducing inflammation, which both contribute to the formation of hyperuricemia and disorders in intestinal homeostasis.

## 5. Nutrition Recommendation, UA–Uric Acid to Reduce Purine and Fructose

So far, the mainstay of dietary treatment of hyperuricemia has been the introduction of a low-purine diet. [66]. This is primarily to avoid products such as meat and seafood, mushrooms, and plant products such as peas, asparagus, and spinach. High-purine products do not include products rich in carbohydrates, such as cereals, groats, rice, or pasta. These products themselves do not contain a harmful amount of fructose, but very often they occur in a processed form, such as ready meals, instant dishes, or bakery products, which are already largely enriched with fructose [67]. Recently, attention has been paid to the fact that hyperuricemia and gout caused by it very often coexist with IR and MetS [68,69]. Due to this correlation, patients with high UA are more likely to develop kidney and cardiometabolic diseases. The traditional low-purine diet, broadly limiting protein-containing products, may be ineffective but also harmful [69]. According to research by Zhu et al. [70], in the US population, as many as 74% of gout patients have hypertension, 71% have kidney problems, 53% are obese, and 26% have developed diabetes. Due to those related facts, the Mediterranean diet or DASH diet is suggested as adequate nutrition. They are characterized primarily by a high content of fresh vegetables and fruits, an increase in fiber, a reduction in saturated fats and an increase in unsaturated fats [71]. Many studies confirmed the effectiveness of the DASH and Mediterranean diets in the case of therapy aimed at lowering blood uric acid and preventing hyperuricemia [72,73,74]. Detailed assumptions of these diets are presented in Table 3.

The Mediterranean diet and the DASH diet, according to research, have a number of positive aspects that, in the long term, reduce the amount of fructose in the diet and lower the concentration of uric acid in the blood [77]. Based on the knowledge that fructose from fruits does not lead to hyperuricemia through fiber, vitamins, flavonols, or trace elements, one way to deduce the harmful effects of fructose is a high-fiber diet [36]. A large amount of fiber from wholegrain products, vegetables, and fruits in the DASH and Mediterranean diets can probably positively affect the effect of fructose and, consequently, reduce the level of UA in the blood. In addition, the significant consumption of plant products, characteristic of the presented diets, increases the supply of vitamins, flavonoids, antioxidants, and antioxidants, and may inhibit increased oxidative stress, platelet aggregation, or inflammation involved in the pathogenesis of metabolic diseases that often coexist with hyperuricemia and hyperuricemia itself [77,78].

**Figure 4:**
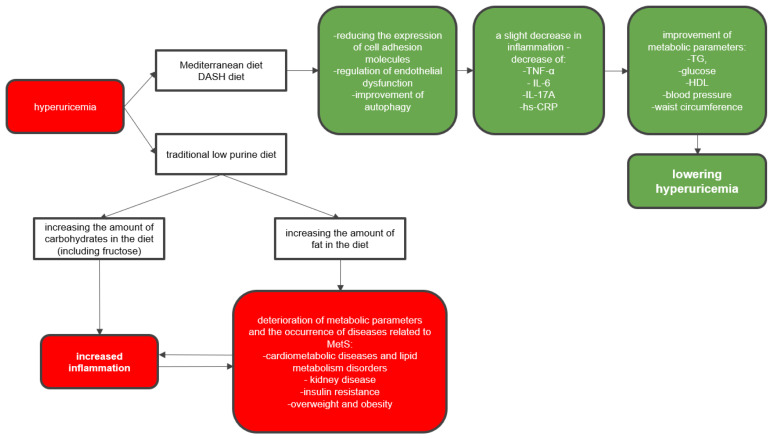
Consequences of improper dietary interventions in patients with hyperuricemia and MetS-related diseases. Following a low-purine diet leads to supplementation of calories with carbohydrates and fats, which may exacerbate MetS-related conditions. The use of an otherwise balanced diet (Mediterranean, DASH) may alleviate the symptoms of MetS and reduce hyperuricemia. Abbreviations: TG—triglycerides; HDL—high density lipoprotein; MetS—metabolic syndrome; TNF-α—tumor necrosis factor α; IL-6—interleukin 6; IL-17A—interleukin 17A; hs-CRP—high sensitivity C-reactive protein; DASH—Dietary Approaches to Stop Hypertension diet.

The assumptions of the mentioned diets have a positive effect on improving metabolic parameters and may significantly contribute to the reduction of hyperuricemia. The consequences of improper dietary interventions in patients with hyperuricemia and MetS-related conditions are presented in Figure 4.

When choosing a Mediterranean diet, we should pay special attention to one aspect that may negatively affect patients with gout. Alcohol in the form of wine is allowed in moderation in this diet. While it has been proven that the type of alcohol matters, and wine does not raise UA levels, it may, like other alcohols, increase the risk of gout attacks. The choice of this ingredient in the diet should be carefully considered by a physician or dietitian and possibly used when the patient has not developed gout [67].

The literature also pays great attention to the role of sugar in developing metabolic diseases and hyperuricemia. Epidemiological data suggest a close association of excessive sugar consumption with conditions such as fatty liver, dyslipidemia, cardiometabolic diseases, IR, type 2 diabetes, and hyperuricemia. Sugar in the diet is necessary and unavoidable, e.g., from eating fruit. The problem seems to be the consumption of excessive amounts of sugar found in sweets, baked goods, sweet drinks, or processed foods, which are increasingly popular on the food market. This has been confirmed by the results of the meta-analysis conducted by Ayoub-Charette et al. [37], which found that unprocessed fruit did not affect the occurrence of hyperuricemia. In addition to adding sucrose, one of the easiest and most convenient ways for producers to add sugar to the product is adding HFCS, which has a negative effect on UA levels [79,80]. A very interesting study was prepared by Walker et al. [81]. They determined the amount of fructose in popular drinks and juices in the United States. Their results suggest that the amounts of free fructose in beverages are higher than previously thought.

To reduce fructose in the diet, specific recommendations may be made to patients, such as:

- choosing water or unsweetened coffee and tea instead of sweetened beverages;
- choosing to eat fruit instead of drinking fruit juices;
- limiting the use of extra sugar during the day, such as sweetening coffee.

The fructose content in selected food products is presented in Table 4.

## 6. Recommendations for Patients Based on a Case Study

We present a proposal of nutritional treatment for patients with gout and metabolic disorders.

Patient A was diagnosed with gout and came to the dietician’s office because he had received pamphlets about the low-purine diet at the hospital, but he is not sure how to implement them. Patient data are presented in Table 5.

Appropriate dietary management based on the management of Patient A is presented in Table 6.

Recommendations for Patient A based on the latest knowledge should be:

- the main goal of the diet is to lower uric acid in order to avoid recurrence of gout;
- laboratory tests indicate the development of metabolic syndrome, which should also be treated with nutrition;
- in order to properly balance the diet, introduce the DASH diet or the Mediterranean diet, with particular emphasis on avoiding high-fructose products, with the exception of fruit and vegetables;
- due to abnormal fasting glucose results and the presence of MetS, it is recommended to test at least fasting insulin to determine whether there is IR;
- if necessary, use pharmacological therapy as prescribed by the doctor.

The patient had an insulin test performed and it turned out that IR had been revealed. Therefore, it was proposed to arrange a diet based on the assumptions of the Mediterranean diet with a slight reduction in carbohydrates in order to achieve better results.

Patient B came to the dietician’s office because of the desire to reduce body weight. During tests ordered by a dietitian after consultation with a family doctor, tests and analyses showed MetS-related disorders, IR, and hyperuricemia. Patient data are presented in Table 7.

Appropriate dietary management based on the management of Patient B is presented in Table 8.

Recommendations for the patient based on the latest knowledge should be the same as for Patient A, except for the amount of carbohydrates in the diet, which due to the presence of IR should be significantly reduced to about 40% [47]. The dietitian made sure that with such a low amount of calories and carbohydrates, the basic carbohydrate requirement for the brain of 130 g/day would be met [84]. For patient B in the prescribed diet, it was 160 g per day.

## 7. Conclusions

A low-purine diet recommended for gout and hyperuricemia is not always an appropriate choice of nutritional treatment. The characteristics of this diet can cause incorrect food choices, leading to excessive fructose consumption. Due to the characteristic metabolism of fructose itself and its metabolites, they can significantly worsen both metabolic parameters and increase the level of uric acid in the body, introducing a vicious circle.

The solution seems to be the Mediterranean diet or the DASH diet, which are characterized by low fructose consumption and an appropriate selection of food products for people with hyperuricemia and gout. It can be concluded that the influence of high levels of UA and fructose on the individual parameters of MetS is significant and requires further research to understand the more precise mechanisms of disease formation and the possibility of planning their treatment.

In the literature, the question arises more often about examining individual sources of fructose and their impact on the induction of hyperuricemia and the MetS syndrome. Attention is also drawn to the need to study unique populations depending on sex, age, or place of confusion to obtain more accurate results and delve more deeply into the individual elements of fructose metabolism. An interesting aspect in this area could be analyzing a comparison of two groups of patients with hyperuricemia on a low-purine diet and a DASH/Mediterranean diet to examine their metabolic parameters and UA levels after carefully planned menus and to examine the effect of consuming large amounts of high-fiber products with the simultaneous use of fructose-rich products other than fruits and vegetables. It could help assess the impact of fiber, polyphenols, or antioxidants on fructose metabolism.

## Figures and Tables

**Table 1:** Diseases associated with symptomatic and asymptomatic hyperuricemia and their mechanism of action.

Symptomatic Hyperuricemia		
>Gout	Stones in the Urinary Tract	[31]
**Asymptomatic Hyperuricemia**		
Chronic kidney disease	deposition of sodium urate crystals oxidative stress endothelial dysfunction fibrosis and inflammation of the kidneys	[7,12,30,32]
Type 2 diabetes mellitus	induction of pancreatic beta cell death weakened insulin signaling	[30,31]
Cardiovascular disease	inducing inflammation endothelial dysfunction proliferation of vascular smooth muscle cells activation of the renin–angiotensin system oxidative stress	[10,30]
Obesity	weakened insulin signaling excess of FFA oxidative stress inflammation	[10,30,32,33]

**Table 2:** Comparison of glucose and fructose metabolism [2,47,48].

Feature	Monosaccharide Sugars	
A Type of Monosaccharide	Glucose	Fructose
glycemic index (GI)	100	23
the main place of deposition of adipose tissue after consuming excess	subcutaneous tissue	visceral adipose tissue
place of metabolism	body cells	- small doses (first order): intestines - higher doses: liver and circulatory system
enzymes involved in metabolism	- glucokinase - phosphofructokinase	- fructokinase
regulation of metabolism	- dependent on phosphofructokinase	- does not depend on phosphofructokinase; - the accumulation of by-products leads to unlimited lipogenesis
serum concentration	- physiologically at a constant level - increases after a meal	- increases with glucose level
insulin dependence	excess causes insulin secretion	lack of phosphofructokinase involvement results in lack of insulin response
impact on appetite	- lowering the level of ghrelin - increasing glucagon-like peptide-1 (GLP-1) - increasing the level of glucose and insulin in the blood	- lowering the level of ghrelin - increasing GLP-1
the effect of metabolism on the body	glucose metabolites prevent excessive phosphorylation	rapid phosphorylation of fructose by the enzymes: - ATP depletion in vascular cells - ATP depletion in the liver - ATP depletion in proximal tubular cells what causes: - transient cessation of protein synthesis production of inflammatory proteins - endothelial dysfunction - oxidative stress

**Table 3:** Assumptions of the Mediterranean diet and the DASH diet, based on [75,76].

Characteristics	Mediterranean Diet	The DASH diet
Carbohydrates [% of total caloric value]	40–45	55
Carbohydrates–characteristics	primary sources: vegetables and fruits	primary sources: whole grains, vegetables, and fruits
Protein [% of total caloric value]	15–18	27
Protein–characteristics	primary sources: lean dairy products, legumes, and fish	primary sources: lean dairy and white meat
Fats [% of total calorific value]	35–45	18
Fats–characteristics	- mainly monounsaturated and polyunsaturated - primary sources: olive oil and nuts	- primary sources: nuts and vegetable oils
Other characteristics	- moderate consumption of red wine [beneficial effect on the lipid profile] - significant consumption of fish, especially fatty sea fish [beneficial effect on the lipid profile]	- high intake of potassium, magnesium, and fiber [influence on glucose metabolism, blood pressure, and insulin action] - organically significant amounts of salt [effects on blood pressure] - organically amount of fat in the diet [improvement of cardiometabolic parameters]

**Table 4:** Fructose content in selected food products based on [81,82,83].

Fructose Content in Selected Food Products	
Product Name	Fructose Content [%] *
Apples	7.6
Bananas	2.7
Raisins, dried	33.8
Corn Flakes	15.48
Pepsi	60.00
7-Up	45.83
Juicy Juice 100% Apple	59.62

* percentage of total sugar in the product.

**Table 5:** Patient A data.

Patient A Data			
Sex	M	HDL-C	48 mg/dL
Age	55 years	Blood pressure	120/75 mmHg
Weight	102 kg	TG	256 mg/dL
Height	178 cm	Fasting glucose	117 mg/dL
Waist circumference	112 cm	UA	8.27 mg/dL

**Table 6:** Appropriate dietary management for Patient A.

Appropriate Dietary Management for Patient A	
carry out, if possible, body composition analysis on a professional analyzer	the test was carried out on the InBody 120 analyzer
checking the content of visceral body fat based on the result from the analyzer	Visceral body fat = 18 *
reading from data from the analyzer the basal metabolic rate (BMR) and determining the total metabolic rate (TMR) based on the physical activity level (PAL) declared by the patient	BMR = 2248 PAL = 1.4 TMR = 3147
determining the appropriate energy deficit and determining the calorific value	caloric content of the diet–2400 kcal **
determination of the percentage composition of macronutrients based on the interview	protein–15% fat–30% carbohydrates–55%

* a high level of visceral adipose tissue additionally indicates the possibility of IR and suggests a slight reduction in the number of carbohydrates. ** a calorific reduction of 750 kcal was applied, which should result in a weight loss of about 0.75 kg/week.

**Table 7:** Patient B data.

Patient B Data			
Sex	K	Blood pressure	148/92 mmHg
Age	48 years	TG	78 mg/dL
Weight	82 kg	Fasting glucose	119 mg/dL
Height	157 cm	UA	8.49 mg/dL
Waist circumference	96 cm	Fasting insulin	32 mLU/L
HDL-C	25 mg/dL	HOMA-IR *	9.4
		QUICK *	0.28

* HOMA-IR and QUICK are among several IR indicators and have been selected by a nutritionist to determine the presence of a condition.

**Table 8:** Appropriate dietary management for Patient B.

Appropriate Dietary Management for Patient B	
carry out, if possible, body composition analysis on a professional analyzer	the test was carried out on the InBody 120 analyzer
checking the content of visceral body fat based on the result from the analyzer	Visceral body fat = 17
reading from data from the analyzer the basal metabolic rate (BMR) and determining the total metabolic rate (TMR) based on the physical activity level (PAL) declared by the patient	BMR = 1488 PAL = 1.4 TMR = 2083
determining the appropriate energy deficit and determining the calorific value	caloric content of the diet–1600 kcal *
determination of the percentage composition of macronutrients based on the interview	protein–30% fat–30% carbohydrates–40%

* a calorific reduction of about 500 kcal was applied, which should result in a weight loss of about 0.5 kg/week.

## Data Availability

All necessary data are included in the paper.
